# Effects of Organoantimony(III) Compounds of Sterically Hindered Bifunctional Tetradentate Ligands on the Reproductive System of Male Rats

**DOI:** 10.1155/MBD.2000.271

**Published:** 2000

**Authors:** R. K. Sharma, M. P. Dobhal, Y. P. Singh, A. K. Rai, R. Sharma, R. Chaudhary, R. Yadav, R. S. Gupta

**Affiliations:** 1 Department of Chemistry University of Rajasthan Jaipur 302 004 India; 2 Department of Zoology University of Rajasthan Jaipur 302 004 India

## Abstract

The antifertility activity of organoantimony(III) complexes PhSb[RC(NC_6_H_4_S)CH_2_(NC_6_H_4_S)CR′] {R' = CH_3_ (R_1_) and R = R' = CF_3_ (R_2_)} derived from corresponding sterically hinlered bifuinctional tetradentate ligands in the male rats was determined. The administration of compounds R_1_ and R_2_ at the dose level of 20 mg/kg. b. wt. siignificantly reduced the weights of testes and epididymides. Auxiliary glands showed a significant reduction after the treatment of compound R_1_ only. Treated animals showed a notable depression of spermatogenesis. The preleptotene spermatocytes were decreased by 76.19 and 47.06; the secondary spermatocytes by 87.4% and 54.87337; and the step-19 spermatids by 72.9 and 46.77% respectively, following the compound R_1_ and R_2_ treatment. Reduced sperm count and motility resulted in 100% negative fertility in both the treated groups. A significant fall in the content of various biochemical parameters of eproductive tissues was observed after R_1_ and R_2_ treatment in comparison to controls.


### EFFECTS OF ORGANOANTIMONY(III) COMPOUNDS OF STERICALLY HINDERED BIFUNCTIONAL TETRADENTATE LIGANDS ON THE REPRODUCTIVE SYSTEM OF MALE RATS

R.K. Sharma[1], M.P. Dobhal[1], Y.P. Singh*[1], A.K.Rai[1], R. Sharma[2], R. Chaudhary[2], R. Yadav[2] and R.S. Gupta[2]

[1] Department of Chemistry, University of Rajasthan, Jaipur (India) 302 004
[2] Department of Zoology, University of Rajasthan, Jaipur (India) 302 004

**Abstract**

The antifertility activity of organoantimony(III) complexes $PhSb[RC(NC_6H_4S)CH_2(NC_6H_4S)CR']$ {R = R' = $CH_3$ ($R_1$) and R = R' = $CF_3$ ($R_2$)} derived from corresponding sterically hindered bifunctional tetradentate ligands in the male rats was determined. The administration of compounds $R_1$ and $R_2$ at the dose level of 20 mg/kg. b. wt. significantly reduced the weights of testes and epididymides. Auxiliary glands showed a significant reduction after the treatment of compound $R_1$ only. Treated animals showed a notable depression of spermatogenesis. The preleptotene spermatocytes were decreased by 76.19 and 47.06; the secondary spermatocytes by 87.4% and 54.8% and the step-19 spermatids by 72.9 and 46.77% respectively, following the compound $R_1$ and $R_2$ treatment. Reduced sperm count and motility resulted in 100% negative fertility in both the treated groups. A significant fall in the content of various biochemical parameters of reproductive tissues was observed after $R_1$ and $R_2$ treatment in comparison to controls.

### Introduction

A large number of antimony(III) compounds have been tested as bactericides[1] and fungicides[2]. The pharmacological activity of antimony compounds has developed ever since the advent of rational chemotherapy [3,4]. A number of antimony compounds have been found most effective against various diseases [5]. Phenothiazines and related compounds with the $-SC_6H_4N-$ moiety are well known to affect the hypothalamous pituitary gonadal axis and thus resulting in a delay in ovulation and menstruation in women [6]. Such type of effects were also observed in rates and dogs [7,8]. The rate of implantation was lowered and reduction in litter size have been reported by some phenothiazine derivatives [9,10]. The survey of the literature revealed that no attention has been paid to the activity of these compounds on the reproductive system of male rats. In the present investigation we are reporting the antifertility activity of organoantimony(III) complexes of sterically hindered bifunctional tetradentate ligands on the male rats.

### Material and Methods

The organoantimony(III) compounds $Ph_3Sb[RC(NC_6H_4S)CH_2(NC_6H_4S)CR']$ where R = R' = $CH_3$ ($R_1$) and R = R' = $CF_3$ ($R_2$) were synthesised by the reaction of $Ph_3Sb$ with the corresponding ligand in 1:1 molar ratio in refluxing benzene. The structure of these compounds have been reported earlier [11]. The organic precursors used for the preparation of these complexes have been synthesised by the condensation of 2-aminothiophenol with corresponding β-diketones ($RCOCH_2COR$).

Wistar rats, weighing 150-180g, obtained from Jamia Hamdard, Hamdard University, New Delhi, were used. Animals were housed in steal cages and maintained under standard conditions (12h light/12 h dark cycle; 25 ± 3°C; 35-60% relative humidity). Rat feed (Hindustan Lever Ltd.) and tap water were provided *ad libitum.*

The route and regimen of treatment were as out lined in table I. On day 61 testes, epididymides, seminal vesicles, ventral prostate, heart, liver and adrenal were removed. The total protein, glycogen and sailic acid were measured [12-14]. Tissues were fixed in Bouin's fluid. Paraffin sections were made and stained with hematoxylin and eosin. The evaluation of cell population dynamics was based on the calculations made for each cell type per cross tubular sections. Various cell componenets were quantitatively analysed. The mating exposure tests of $R_1$ and $R_2$ treated male groups were performed from day 55 to day 60. The mated females were separated to note the implantation sites on day 16th of pregnancy through leparotomy. The results were analysed using student "t" test.

### Results and discussion

The administration of $R_1$ at the dose level of 20 mg / kg b. wt. caused the significant reduction in the body weights of treated rats. However, $R_2$ did not cause any significant change in body weights. The weights of testes ($P < 0.001$), epididymides ($p < 0.001$), seminal vesicle ($p < 0.01$) and ventral prostate ($p < 0.001$) were reduced significantly following the $R_1$ treatment. Whereas $R_2$ treatment only reduced the weight of testes and epididymides. The number of step-19 spermatids were decreased by 72.9 and 46.77% following $R_1$ and $R_2$ administration, respectively. The population of preleptotene spermatocytes were decreased by 76.19

and 47.06 in $R_1$ and $R_2$ treated rats. The secondary spermatocytes were decreased by 87.4% and 54.8%, respectively (table-II).

**Table-I. Effects of $R_1$ and $R_2$ on the body wt. and the organ weights.**

| Treatment | Body Weight(g) | Organ Weight (mg/100 g b.wt.) | | | |
|---|---|---|---|---|---|
| | | Testes | Epididymides | Seminal Vesicle | Ventral Prostate |
| Gr.1 | 240±1.4 | 1345±4.7 | 529.5±11.2 | 605.7±9.5 | 308.5±2.02 |
| Gr.2 | 165**±7 | 1031.53**±45 | 293.61**±9.14 | 566.8*±7.0 | 123.82**±11.01 |
| Gr.3 | 218±10$^{ns}$ | 1065±88* | 369**±13.4 | 607±25$^{ns}$ | 336.72±14.0$^{ns}$ |

ns : non significant, All figures ± SEM, Levels of significance : *$P < 0.01$, **$P < 0.001$

Gr.1 Rat receiving vehicle (olive oil 0.2 ml/day) i.p. for 60 days

Gr.2 Rat treated with $R_1$ (20 mg/kg b.wt.) i.p. for 60 days

Gr.3 Rat treated with $R_2$ (20 mg/kg b.wt.) i.p. for 60 days

**Table-II. Testicular cell population dynamics following $R_1$ and $R_2$ Treatment**

| | Testicular cell counts (number / 10 cross section) | | | | | |
|---|---|---|---|---|---|---|
| | Sertoli cell | Spermatogonia | Preleptotene | Pachytene | Sec.spermat ocytes | Step-19 spermatids |
| Gr.1 | 2.81 ±0.02 | 6.87±0.65 | 19.95±1.9 | 29.29±0.73 | 48.1±3.8 | 29.8±2.8 |
| Gr.2 Percent deviation | 1.75 ±0.07** | 2.27±0.1** (-) 66.95 | 4.75±0.45** (-)76.19 | 15.65±1.2** (-)46.56 | 6.06±1.9** (-)87.40 | 8.075±1.1** (-)72.9 |
| Gr.3 Percent deviation | 2.6 ±0.15$^{ns}$ | 3.8±0.4* (-)44.68 | 10.56±1.2* (-)47.06 | 25.04±1.1* (-)14.51 | 21.74±2.5* (-)54.8 | 15.86±1.7* (-)46.77 |

ns : non significant, All figures ± S.E.M.,

Levels of significance: *$P < 0.01$ Compared with Control, **$P < 0.001$ Compared with controls

As shown in table III the protein content of testes, epididymides and ventral prostate were reduced significantly ($p < 0.001$) following $R_1$ treatment at the dose level of 20 mg / kg b. wt. as compared with control. $R_2$ treatment bring about the reduction of protein contents of cauda epididymides, sailic acid contents of the testes, epididymides and auxiliary glands (seminal vesicle and ventral prostate) were depleted in both the treated groups. Glycogen contents of testes were decreased significantly ($p < 0.001$) in comparison with controls. Fructose in seminal vesicle was also declined.

$R_2$ as shown in table IV the $R_1$ and $R_2$ treated rats showed significant ($p < 0.001$) reduction in the sperm concentration of testes and epididymides. The motility of the cauda epididymal sperm was also reduced significantly ($p < 0.001$). Both the treatments reduced the fertility of male rats by 100%.

Administration of $R_1$ and $R_2$ for 60 days to male rats brought about a significant loss in testes weights, which is mostly related to the nubmer of spermatids and spermatozoa present in the tissue. The reduced testicular weights also indicative of wide spread damage [15]. Low cauda epididymal sperm count, presence of non-motile spermatozoa and significant reduction in epididymal weights imply that these compounds induced infertility might be caused by several factors [16] including the oxidative phosphorylation uncoupling [17].

The reduction in sperm density and motility in cauda epididymides is of importance with regard to fertilization [18]. Reduction in number of sertoli cells following $R_1$ treatment adversely affects the cell cycle kinetics and influence both spermatogonia and praleptotene spermatocytes [19]. Depletion in protein, sailic acid contents of testes and epididymides and auxiliary glands and testicular glycogen and fructose in seminal vesicle reflects the antispermatogenic effects of the $R_1$ and $R_2$ compounds. The results demonstrate that the compound $R_1$ is more potent than compound $R_2$.

Spectral evidences for both the compounds suggest [11] the presence of a pentacoordinated antimony(III) atom in the pseudo-octahedral complexes, the geometry of which is due to the presence of lone pair of electrons is. Spectral evidences also suggest a distortion in the basal plane of the complexes.

**Table-III. Effect of $R_1$ and $R_2$ Treatment on biochemical parameters of male rat.**

| | Protein(mg/gm) | | | | Sialic acid (mg/gm) | | | | Glycogen (mg/g) | Fructose (mg/g) | Ascorbic acid (mg/gm) |
|---|---|---|---|---|---|---|---|---|---|---|---|
| | Testes | Cauda Epididymis | Seminal Vesicle | Ventral Prostate | Testes | Cauda Epididymis | Seminal Vesicle | Ventral Prostate | Testes | Seminal Veside | Adrenal |
| Gr.1 | 173.88 ± 5.31 | 211.14 ±1.13 | 192.74 ± 2.94 | 171.12 ± 1.06 | 4.67 ± 0.01 | 6.27 ± 0.05 | 5.26 ± 0.04 | 4.82 ± 0.02 | 2.16 ± 0.4 | 4.66 ± 0.2 | 3.30 ± 0.2 |
| Gr.1 | 155.53* ± 2.12 | 148.87** ± 3.85 | 182.2$^{ns}$ ± 5.13 | 155.54** ± 3.8 | 3.57** ± 0.01 | 3.63** ± 0.05 | 3.39** ± 0.05 | 3.63** ± 0.01 | 1.48** ± 0.02 | 2.66 ± 0.1** | 3.18$^{ns}$ ± 0.04 |
| Gr.3 | 168.86$^{ns}$ ± 2.5 | 153.3** ± 5.8 | 191.09$^{ns}$ ± 1.28 | 166.64$^{ns}$ ± 1.28 | 3.66** ±0.05 | 3.66** ± 0.017 | 3.54** ± 0.034 | 3.66** ± 0.03 | 1.61** ± 0.02 | 3.99 ± 0.1* | 3.44 ± 0.1$^{ns}$ |

ns : non significant, Values are mean ± S.E. of six determinations,
Levels of significance : *P < 0.01, **P < 0.001

**Table-IV. Sperm motility, concentration and fertility after $R_1$ and $R_2$ treatment**

| Treatment | Sperm motility (Cauda epididymes) | Sperm density (million/ml) | | Fertility (%) |
|---|---|---|---|---|
| | | Testes | Cauda epididymides | |
| Gr.1 | 68.02 ± 2.78 | 4.66 ± 0.59 | 44.21 ± 2.3 | 100 (+ ve) |
| Gr.2 | 9.78 ± 2.64** | 1.15 ± 0.4** | 4.30 ± 0.17** | 100 (-ve) |
| Gr.3 | 33.89 ± 4.0** | 2.35 ± 0.2* | 8.42 ± 1.6** | 100 (-ve) |

All figures ± SEM, Levels of signficance : *P < 0.01, **P < 0.001

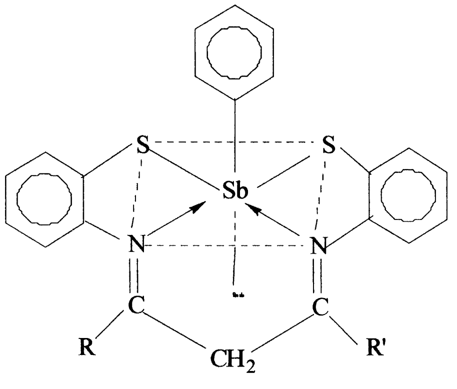

Fig. 1 Structure of $PhSb[RC(NC_6H_4S)CH_2(NC_6H_4S)CR']$

It is evident from the above disussion that the antifertility activity of the compound ($R_1$) is more than the compound $R_2$. It is a well known fact that the presence of halogen atom in the compound enhances its antifertility activity[20,21]. But in the present study the hexafluoro derivative $R_2$ was found to be less active than the non-fluorirated derivative. It might be possible that compound $R_2$ may have a positive effect on male reproductive system. This may be verified by carrying out the toxicological effect of both the compounds. The study is in progress and the results will be published in due course of time.

**Acknowledgement**

One of the authros (R.K. Sharma) is thankful to University Grants Commission, New Delhi for financial assistance.

**References**

1. Fr. Demande, 2, 013, 455, 1970; CA 74, 1971, 3727.
2. J.D. Curry, U.S. Pat., 3, 833, 565, 1974; CA 82, 1974, 88069
3. B.M. Gatehouse, *Chem. Commun.*, 1969, 948.
4. C.A. McAuliffe, '*Comprehensive Coordination Chemistry*' Editor in chief, G. Wilkinson, **3**, 1987, pergmon Press (U.K.).
5. J.L. Wardell, '*Comprehensive Organometallic Chemistry*', **1**, 1982, 681; Eds. E. W. Abel, F.G.A. Stone and G. Wilkinson, Pergmon Press (U.K.); J.L. Wardell, '*Comprehensive Organometallic Chemistry*', **2**, 1995, 321; Ed. A. G. Davis, Editor in Chief, E. W. Abel, F.G.A.Stone and G. Wilkinson, Pergmon Press (U.K.).
6. T. Miyato, K. Takahama, T. Irie and K. Uekama, *In Bioactive Molecules* (Vol. 4), Chapter II, Ed. R.R. Gupta, Elsevier press, Amserdam.

7. L. Julou, R. Dcrot, P. Ganter, R. Maral P. Populaire, J. Durel, E. Hattric, J. Myon, S. Pascal and J. Pasquet, *Proc. Eur. Soc. Study Drug Toxical.*, **9**, 1968, 11.
8. R. Degkwitz, C. Heushgem, L.E. Hollister J. Jacob, L. Julou, P.A. Lambert, R. Marsbom, W. Meier-Rung, W.K.A. Schaper and H.C. Tuchmann-Duplessin, '*Toxicity and Side Effect in Man and in the Laboratory Animal*' in D.P. Bobon P.A.J. Janssen and J. Bobon (Ed.), *The Neuroleptics, Kargar Base,* 1970, 671.
9. J.H. Ravina, *Presse Med.*, **72**, 1964, 3057.
10. F.E.Hrrington, R.G. Eggert, R.D. Wlbur and W.H. Linkenheimer, *Endocrinology*, 1966, 1130.
11. R.K. Sharma, Y.P. Singh and A.K. Rai, *Synth. React. Inorg. Met. Org. Chem.*, 2001 (Accepted)
12. O.H. Lowry, M.T. Rosebrough, A.L. Farr and R.J. Randall, *J. Biochem.*, **193**, 1951, 265.
13. R. Montgomery, Archives Biochem. *Biophysics,* **57**, 1957, 378.
14. L. Warren, *J. Bio. Chem.,* **234**, 1959, 1971.
15. A.B. Keel and T.O. Abney, *Endocrinology*, **107**, 1980, 1226.
16. S.K. Saksena and R.A. Salmonsue, *Fertil. Steril.*, **37**, 1982, 686.
17. Y.B. Kee and W.W. Tso, *Int. J. Fert.*, **27**, 1982, 42.
18. J.M. Bedford, *Biol. Reprod.*, **28**, 1983, 108.
19. A. Gasinska and S. Hill, *Neoplasma*, **37**, 1990, 357.
20. J. M. Robson, A. Schonberg, *Nature*, **22**, 1942, 150.
21. V.N. Pathak, R.K. Chaturvedi, S. Sharma M. Jain and K.C. Joshi, *Pharmazie*, **323**, 1993, 48.

**Received: December 13, 2000 - Accepted: January 19, 2001 -
Received in revised camera-ready format: January 23, 2001**

